# Bruns Syndrome: a deadly sign

**DOI:** 10.11604/pamj.2015.22.229.8140

**Published:** 2015-11-11

**Authors:** Mohammed Yassaad Oudrhiri, Nabil Raouzi

**Affiliations:** 1 Neurosurgery Department, Al Farabi Regional Hospital Center, Oujda, Morocco

**Keywords:** Bruns syndrome, colloid cyst, hydrocephalous

## Image in medicine

Bruns syndrome was first described in 1902, as a sudden onset of severe headaches and vomiting associated to a vestibular syndrome provoked by abrupt change in head position. It is related to an episodic obstructive hydrocephalous caused by an intraventricular mass that acts like a ball-valve mechanism. A 52 years old man was admitted to the intensive care unit for a sudden onset coma. His Glasgow Coma Scale was 4/15 with fixed dilated pupils. He has been experiencing paroxystic episodes of headaches, vomiting and vertigo for 6 months that went undiagnosed.Diagnoses include acute hydrocephalous, syncopes, and stroke. The CT scan showed a globally iso-dense rounded mass of the anterior roof of the third ventricle, with hyperdense areas suggestive of hemorrhage, obstructing both foramen of Monro and causing acute hydrocephalous. The patient underwent an urgent placement of an external ventricular drainage; the intracranial pressure was above 25mmHg, and the CSF gin-clear.Unfortunately, the patient did not survive this episode and died 24hours later. Colloid cysts are rare, benign, and curable lesions located at the anterior part of the roof of the third ventricle, and contain colloid material. Their clinical presentation varies from incidentally found cysts to sudden death.Rapid deterioration may also be caused by an intrinsic hemorrhage. Such condition has only been reported in the literature in 16 publications. Through this observation we would like to bring attention concerning this syndrome that may be preceding fulminant and lethal deterioration from a benign and surgically curable lesion.

**Figure 1:**
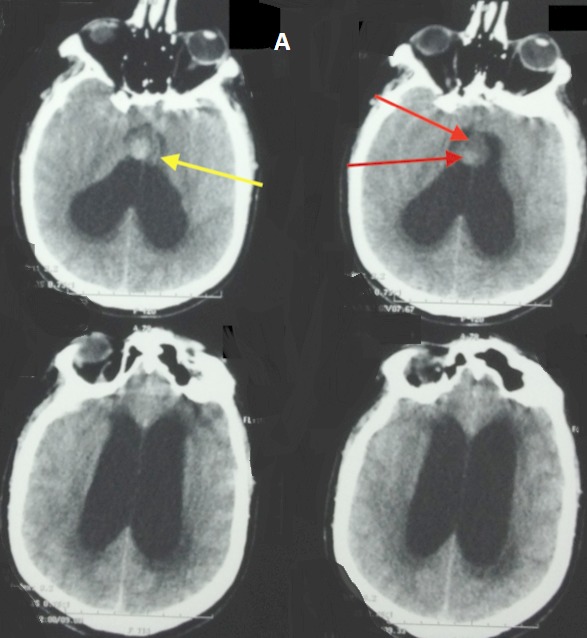
Hemmorhagic colloid cyst with acute hydrocephalous

